# Assessment of mGluR5 KO mice under conditions of low stress using a rodent touchscreen apparatus reveals impaired behavioural flexibility driven by perseverative responses

**DOI:** 10.1186/s13041-019-0441-8

**Published:** 2019-04-11

**Authors:** Jisoo Lim, Eosu Kim, Hyun Jong Noh, Shinwon Kang, Benjamin U. Phillips, Dong Goo Kim, Timothy J. Bussey, Lisa Saksida, Christopher J. Heath, Chul Hoon Kim

**Affiliations:** 1 Department of Pharmacology, BK21 PLUS Project for Medical Science, Brain Research Institute, Yonsei University College of Medicine, 50-1 Yonsei-ro, Seoul, 03722 Republic of Korea; 2 Department of Psychiatry, Institute of Behavioural Science in Medicine, BK21 PLUS Project for Medical Science, Yonsei University College of Medicine, 50-1 Yonsei-ro, Seoul, 03722 Republic of Korea; 3 Department of Psychology and MRC/Wellcome Trust Behavioural and Clinical Neuroscience Institute, University of Cambridge, Downing Street, Cambridge, CB2 3EB UK; 4 Molecular Medicine Research Laboratories, Robarts Research Institute & Department of Physiology and Pharmacology, Schulich School of Medicine & Dentistry, The Brain and Mind Institute, Western University, London, ON Canada; 5 School of Life, Health and Chemical Sciences, The Open University, Walton Hall, Milton Keynes, MK7 6AA UK; 6 Severance Biomedical Science Institute, Yonsei University College of Medicine, Seoul, 03722 South Korea

**Keywords:** mGluR5, Behavioural flexibility, Perseveration, Reversal learning, Extinction, Progressive ratio schedule

## Abstract

Genetic and pharmacological manipulations targeting metabotropic glutamate receptor 5 (mGluR5) affect performance in behavioural paradigms that depend on cognitive flexibility. Many of these studies involved exposing mice to highly stressful conditions including electric foot shocks or water immersion and forced swimming. Because mGluR5 is also implicated in resilience and stress responses, however, apparent impairments in inhibitory learning may have been an artifact of manipulation-induced changes in affective state. To address this, we present here a characterization of cognitive flexibility in mGluR5 knockout (KO) mice conducted with a rodent touchscreen cognitive assessment apparatus in which the animals experience significantly less stress.

Our results indicate a significant reversal learning impairment relative to wild-type (WT) controls in the two-choice Visual Discrimination-Reversal (VDR) paradigm. Upon further analysis, we found that this deficit is primarily driven by a prolonged period of perseveration in the early phase of reversal. We also observed a similar perseveration phenotype in the KO mice in the Extinction (EXT) paradigm. In addition, mGluR5 KO mice show higher breakpoints in the touchscreen Progressive Ratio (PR) and altered decision making in the Effort-related Choice (ERC) tasks. Interestingly, this impairment in PR is an additional manifestation of an increased propensity to perseverate on the emission of relatively simplistic behavioural outputs.

Together, these findings suggest that under conditions of low stress, mGluR5 KO mice exhibit a pronounced perseverative phenotype that blunts cognitive flexibility.

**Electronic supplementary material:**

The online version of this article (10.1186/s13041-019-0441-8) contains supplementary material, which is available to authorized users.

## Introduction

Behavioural flexibility is a key cognitive ability required for effectively addressing the demands of a constantly changing environment. The mGluR5 subtype of metabotropic glutamate receptors is involved in this cognitive function [1–4]. The studies characterizing this relationship have assessed rodents in which mGluR5 receptor expression or function has been manipulated with genetic or pharmacological tools in reversal learning or extinction paradigms. Notably, their behaviours were measured in every study under high stress conditions, for example, using classical (Pavlovian) fear conditioning or water maze escape paradigms [1–5].

Exposure to aversive stimuli such as foot shock and forced swim can induce stress responses that affect learning, planning [6–10] and reward responsiveness [11, 12], making it difficult for paradigms that use aversive stimuli to discriminate between stress susceptibility and cognitive rigidity. This is particularly important in studies of mGluR5, because mGluR5 is critical in resilience and the responses of mice to stressful stimuli [13, 14]. An mGluR5-dependent impact on overall affective state could, therefore, contribute indirectly to any observed change in behavioural flexibility. We hence wanted to assess the effects of mGluR5 manipulation on cognitive flexibility in a relatively low-stress operant context to provide further insight into the role this receptor plays in this process.

To do this, we evaluated the cognitive flexibility of mGluR5 KO and WT littermates using a rodent touchscreen cognitive assessment apparatus [15, 16]. This platform exclusively uses appetitive reinforcement to avoid stress-related confounding effects. Animals are assessed in sound-attenuated, darkened behavioural chambers to which they are thoroughly habituated. This system is also automated such that the experimenter does not handle the animals during testing, thus minimizing the stress associated with experimenter-animal interactions. The use of computerized task delivery, data recording, and analysis also ensures full standardization between chambers, as well as robust paradigm stability between sessions and the elimination of experimenter/scorer bias within and across studies [16–20]. These attributes contribute to high data reproducibility that enables direct comparisons of studies both within and between research groups [19, 21].

Here, we report that under conditions of low stress, the absence of mGluR5 yields impairments in cognitive flexibility in both the Visual Discrimination Reversal (VDR) and Extinction learning (EXT) paradigms. Further analysis of these data identified a pronounced perseverative responding phenotype in the mGluR5 KO mice. This trait also manifested itself as apparent insensitivity to the lack of reward on a touchscreen Progressive Ratio (PR) schedule of responding, and disrupted reward-related decision making in the touchscreen Effort-related Choice (ERC) paradigm.

## Materials and methods

### Animals

All experiments were conducted with male mGluR5 KO mice [22] on a C57BL/6J background. WT and mGluR5 KO littermates were bred at Yonsei University through heterozygous-heterozygous mating. Offspring were genotyped by PCR analysis.

The behavioural testing presented here used four genetically modified animal cohorts: cohort 1 (*n* = 7 for WT and *n* = 7 for KO) was tested on VDR at 20–24 weeks of age at the outset of training; cohort 2 (*n* = 8 per genotype) was tested on EXT at 16–21 weeks of age at the outset of training; cohort 3 (*n* = 10 per genotype) was tested on fixed ratio (FR), PR and ERC at 20–28 weeks of age at the outset of training; cohort 4 (*n* = 10 per genotype) was tested on EXT and subsequently on PR at 25–36 weeks of age at the outset of training.

For the behavioural pharmacology study using the mGluR5 antagonist MTEP, male C57BL/6J mice (*n* = 16) (Central Lab, Animal Inc., Seoul, Korea) were purchased at 10 weeks of age and given a 7-day facility acclimatization period comprising only routine husbandry until the study began.

All mice were handled and weighed daily to establish free-feeding weights. They were housed in groups of 2–4 per cage in a humidity- and temperature-controlled, specific pathogen-free environment (lights on at 8:00 am) in the Yonsei University College of Medicine Animal Care Facility. All animal experiments were approved (No. 2015–0287 and 2018–0278) by the Animal Care Committee of Yonsei University College of Medicine using US National Institutes of Health Guidelines. Cages were changed by experimenters once a week with food and water available ad libitum.

For food restriction, the daily provision of chow pellets was adjusted to yield daily weight reductions of no more than 5% from the previous day. Weights were measured daily throughout the study and chow provisions were adjusted to maintain the mice at approximately 85% of free-feeding weight throughout the experiment. Once stable food restriction was achieved, animals were trained in the touchscreen chamber once a day for 5–7 days a week, during the light phase of the cycle.

### Reward and drugs

Strawberry-flavored milk (SM; SeoulMilk Dairy Cooperative, Seoul, Korea) was used as the reinforcer in this study. The nutritional parameters and efficacy of SM in touchscreen tasks were previously described [21].

3-((2-methyl 1,3-thiazol-4-yl)ethynyl) pyridine hydrochloride (MTEP hydrochloride, Tocris Bioscience) was dissolved in 1% Tween 80 (vol/vol) and 99% sterile saline. Either MTEP (10 mg per kg, i.p.) or vehicle was administered as a series of triple injections; 23 h, 15 h and 1 h prior to the first extinction session. Then, a daily single injection was administered 1 h before being put in the touchscreen chambers beginning with session two until the end of the study.

### Apparatus

Testing was conducted in standard Bussey-Saksida mouse touchscreen chambers (Campden Instruments Ltd., Loughborough, UK) that have been described in detail elsewhere [15, 16, 18, 20, 23]. These consist of an operant arena housed within a sound- and light-attenuating chamber. The trapezoid-shaped arena consists of a stainless-steel floor surrounded by reinforced plastic walls with a touchscreen (12.1-in., screen resolution 800 × 600) mounted at one end and a reward magazine at the other. Infra-red beams run across the floor of the chamber to monitor locomotor activity with the front beam approximately 6 cm from the screen and the rear beam approximately 3 cm from the magazine. An IR beam in the magazine monitors head entries, which are used to initiate trials. The magazine also contains a light-emitting diode (LED), which is lit to signal that a trial can be initiated by head entry or that a liquid reward has been delivered. Reward delivery in this study consisted of an 800 ms activation of the pump built into the behavioural chamber to yield delivery of 20 μL of SM to the magazine. Upon reward collection, the LED is turned off, and the next trial can be initiated. Black plastic masks are placed in front of the touchscreen to provide defined response locations and to minimize non-specific interactions with the screen by the animals. In this study, a 2 × 1 mask (Campden Instruments, Ltd) was used for VDR. This mask contains a row of 2 square (7 × 7.5 cm) response windows, spaced 0.5 cm apart. A 3 × 1 mask (Campden Instruments, Ltd) was used in the EXT. This consists of a row of 3 square (7 × 7 cm) response windows, spaced 0.5 cm apart. For the FR, PR, and ERC tasks, a 5 × 1 mask (Campden Instruments, Ltd) consisting of a row of 5 square (4 × 4 cm) response windows, spaced 1 cm apart across the mask situated at 1.5 cm above the floor was used. In these two paradigms, the visual stimuli appeared only in the central response location.

### Touchscreen behaviour training

The animals were first habituated to the behavioural chambers in 2 consecutive 20-min sessions during which IR beam breaks at the touchscreen were recorded to track locomotor activity but no stimuli were presented and no programmed behavioural consequences were delivered. To aid habituation, 200 μL of SM was delivered to the magazine at the beginning of each session. Criterion for completing these sessions required mice to consume the available SM in both sessions.

The initial behavioural training session comprised a 60-min session to associate touchscreen visual stimulus offset with reward delivery. For the FR, PR, ERC and EXT tasks, a white square (4 × 4 cm) was presented in the central response window for 30 s. For the VDR task, 1 of 20 randomly shaped, black and white stimuli (approximately 5 × 5 cm) was displayed in one of the two response windows, with the location selected pseudorandomly such that no image would be displayed in the same window more than 3 times in a row. Upon stimulus offset, 20 μL of SM was delivered with a tone (1000 ms, 3 kHz) and magazine illumination. After reward collection, the magazine light was turned off and a 5-s inter-trial interval (ITI) (or 20-s ITI for reversal) was imposed before the next trial could commence. If the stimulus was touched while illuminated, it was immediately turned off and triple reward delivery (60 μL) was provided. Criterion for this stage required animals to collect 30 rewards in a session.

In the next stage of training, the presented stimulus remained on the screen until touched. This too was rewarded with 20 μL SM, accompanied by a tone and magazine illumination. Training in this stage continued until animals completed 30 trials within 60 min.

For animals training for VDR, an additional stage of training is necessary. This requires animals to touch a visual stimulus to receive a 20 μL SM reward, but responses in the window without the visual stimulus trigger a time-out in which the chamber house light is illuminated. After this time-out and an ensuing ITI, the magazine light is illuminated to signal that a head entry will initiate a new trial. Criterion for this stage required mice to achieve more than 77% correct responses within 30 min for 2 consecutive days.

### VDR task

#### Acquisition

Mice were presented with two brightness-matched stimuli, one of which was correct (S+) and the other incorrect (S−). A nose poke to S+ resulted in a tone, magazine illumination and a 20 μL reward delivery. A nose poke to S− resulted in house light illumination and a time-out. Each session consisted of 30 trials with pseudorandom selection of stimulus location presentation and a 20-s ITI. Animals were required to achieve 80% or more correct choices for 2 consecutive days to achieve the performance criterion.

#### Reversal

After discrimination acquisition, all mice performed 3 further sessions to reinforce the reward contingencies and ensure stable baseline performance. On the following day, the S+ and S− contingencies were reversed. The reversal phase continued until mice reached more than 80% correct responses for 2 consecutive sessions. The early phase of reversal learning was analyzed through a summation of all the errors in each session (30 - correct trials) before each individual mouse reached 50% accuracy [7, 10, 24, 25].

### EXT task

#### Acquisition

In this phase, a single stimulus was presented in the center response window of the 3-hole mask. A nose poke to the stimulus was rewarded with SM delivery accompanied by magazine illumination without tone delivery. Responses in the blank response windows had no programmed consequences. Each session consisted of 30 trials (with 5-s ITIs). Training continued until the animals completed 30 trials in 12.5 min for 5 consecutive days. The training criterion was adjusted to the completion of 30 trials in 6 min for 5 consecutive days with tone delivery for stronger acquisition learning so that multi-session analyses could be performed in the following EXT task.

#### Extinction

During the extinction phase, the stimulus was again displayed in the middle window. The stimulus disappeared when the animal touched the window (response) or after 10 s (omission) with no reinforcement provided. Each session terminated after 30 trials had been performed (~ 10 min). The number of stimulus responses made in the first 3 min of the session on day 1 was evaluated for single session analysis. For multi-session analyses, the extinction phase was continued until animals attain a criterion of 77% omissions (less than 23% responses) for 2 consecutive days.

### FR/PR task

The general FR task procedure in the touchscreen was previously described [15, 23]. Following initial training, animals progressed to FR training. Mice were trained to respond to a white square stimulus presented in the central response window. All animals were trained in FR1, in which a single touchscreen response was required to earn a single SM reward, followed by FR2 (two responses per reward) and FR3 (three responses per reward). Touching the stimulus during the FR2 or FR3 schedules resulted in a brief (500 ms) removal of the screen stimulus and the delivery of a ‘chirp’ tone (10 ms, 3 kHz). Each FR training session was limited to either the completion of 30 trials for FR1, 15 trials for FR2, 10 trials for FR3 or a 60-min time. Criterion was defined as completion of 30 touch responses in a single session.

Once criterion was reached, the mice progressed to the more demanding FR5 schedule, in which a single reward requires 5 correct touchscreen responses and 150 touch responses are required to reach criterion. Three consecutive sessions of FR5 were performed to ensure animals developed high selectivity for the target location, avoiding excessive responses in the other four ‘blank’ locations, and to ensure sustained response levels. Following FR5 training, the animals progressed to two sessions of unrestricted FR5 (FR5-UC) with no maximum trial limit across each 60 min session.

Following FR assessment, the mice were transferred to the PR schedule. The task parameters under the PR schedule were identical to those used in the FR sessions, except that upon completion of each trial, the reward response requirement was increased on a linear + 4 basis (i.e., 1, 5, 9, 13, etc.). If no response to the screen or no magazine entry was detected within 5 min, the sessions were automatically terminated. Task performance was evaluated by monitoring the breakpoint, which was defined as the number of target responses emitted by an animal in the last successfully completed trial of a session. Other evaluated performance parameters included blank touches (responses at the 4 non-target screen locations), reward collection latency (time between completion of the final target touch of a trial and entry to the reward magazine for reward collection), and the rate of front and rear IR beam breaks.

### ERC task

Animals were trained on FR8, 16 and 32 for 5 consecutive sessions of each work requirement using the FR task parameters detailed previously, with the exception that three pellets of standard laboratory chow were weighed and scattered randomly across the floor of each chamber prior to the start of each session. Upon session completion, mice were immediately removed from the chambers and any remaining pellets and pellet fragments on the floor of the arena or in the waste collection tray were collected and weighed to calculate chow consumption. Task performance of each operant work requirement was evaluated in terms of the volume of milk consumed which is linearly related to the number of trials completed.

### Chow and SM consumption assessment

Feeding behaviour in the presence of large quantities of chow or SM was measured to assess the levels of chow and SM consumption under effort-free conditions. Standard laboratory chow or SM were independently provided to individually caged mice and consumption was monitored for 12 h across 2 days. This procedure was conducted during the light phase of the 12 h light-dark cycle in a quiet behaviour testing room.

### Chow versus SM preference assessment

Free access home cage preference tests were performed to determine whether mice assign different relative values to the two reward options provided in the ERC paradigm (chow vs. SM) in the absence of the effort-related requirements. This procedure was conducted in clean standard housing cages in a quiet behaviour testing room. Each cage was prepared with a bowl attached to the center of the floor. Four weighed standard laboratory chow pellets were randomly placed on the floor of the cage and the bowl was filled with SM. Mice were then allowed 60 min to freely consume either substance. After the session ended, the mice were returned to their home cages and the remaining chow and SM were weighed to measure consumption. This procedure was repeated for 5 days.

### Statistical analysis

Behavioural data analysis was conducted with GraphPad Prism version 7 (GraphPad Software, Inc., La Jolla, CA, USA). Differences between means were assessed with a *t* test or analysis of variance (ANOVA) as appropriate. Two-way ANOVA or two-way repeated measures (RM) ANOVA was conducted to assess the main effects of genotype and other conditions (operant requirement or session) and the genotype by condition interactions. Whenever the ANOVA showed significant effects, the Bonferroni post hoc test was used. The within-session response rate analysis in FR5-UC and PR4 was conducted as previously described [21, 26, 27]. The total response time for each trial was converted to a response rate (responses per minute) and fitted with the equations y = b*(x)^2 + a for FR and y = a^(−b*x) for PR using non-linear least squares regression. Using these fits, we obtained predicted values for the peak response rate (a) and decay rate (b) for each individual animal. All data were presented as means ± standard error of the mean (SEM). The significance level was set at *p* < 0.05.

## Results

### mGluR5 KO mice exhibit impaired reversal in the touchscreen two-choice VDR task

**Fig. 1:**
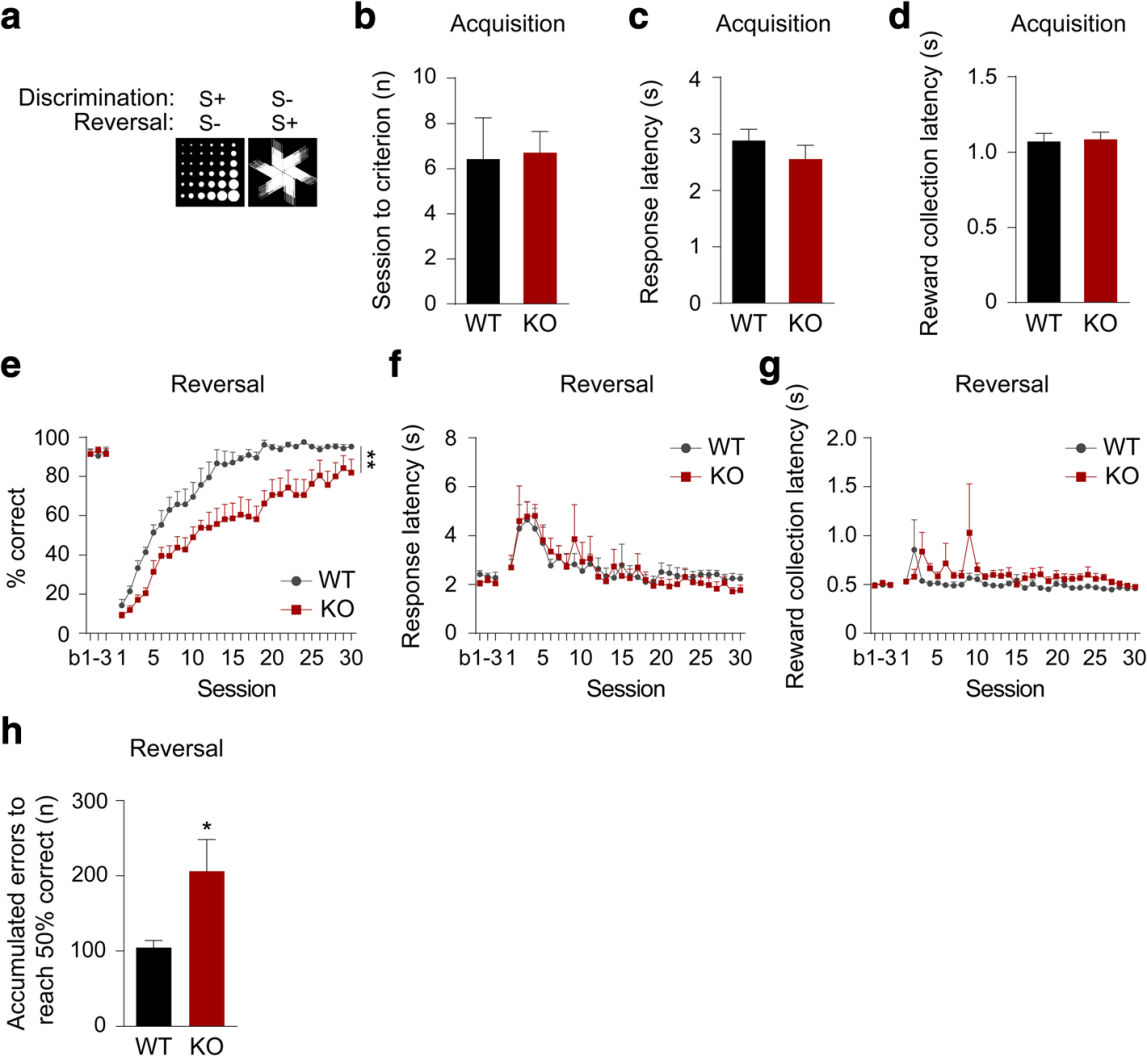
mGluR5 KO mice are impaired in the reversal phase of the touchscreen VDR task. **a** Images of the ‘marble’ and ‘fan’ stimuli used in the VDR task. **b** Mean number of sessions required to reach the VDR acquisition criterion (> 80% correct responses for 2 consecutive days). **c**-**d** Mean response latency and mean reward collection latency across VDR acquisition sessions. **e** Percentage of correct during 3 baseline training sessions (b1-b3) and following contingency reversal sessions (Two-way RM ANOVA, main effect of genotype; **p = 0.002). **f**-**g** Response latency and reward collection latency through the reversal sessions. **h** Accumulated errors to reach 50% correct (early phase) in reversal learning (Unpaired *t* test; *p = 0.037). WT group *n* = 7 and mGluR5 KO group *n* = 7. All data are presented as means ± s.e.m

To evaluate the importance of mGluR5 in cognitive flexibility under low-stress conditions, we assessed a cohort of mGluR5 KO mice and WT littermates in the touchscreen two-choice VDR task using the ‘fan’ and ‘marbles’ stimuli (Fig. 1a) [16] . For the acquisition phase of the VDR task, we found no difference between genotypes in the number of sessions required to reach criterion (Fig. 1b), suggesting both genotypes show comparable performance in simple perceptual discrimination and stimulus-reward association learning. We also found no between-genotype differences in either response latency (Fig. 1c) or reward collection latency (Fig. 1d) during the acquisition phase.

When the stimulus reward contingencies were reversed, however, it became clear that mGluR5 KO mice are impaired relative to WT (Two-way RM ANOVA, main effect of genotype; F(1,12) = 14.9, *p* = 0.002, main effect of session; F(29,348) = 56.4, *p* < 0.001, genotype x session interaction; F(29,348) = 1.32, *p* = 0.128) (Fig. 1e) in the absence of any change in response latency (Fig. 1f) or reward collection latency (Fig. 1g).

Perseveration is a term generally used to indicate abnormal repetitive behaviors and is observed in various neuropsychiatric illnesses [28]. As a general term, it covers several phenomena, including stuck-in-set perseveration, recurrent perseveration, and continuous perseveration [29]. Further analysis of the early phase (Accuracy < 50%) of reversal learning revealed that mGluR5 KO mice are significantly impaired relative to WT in the early phase where perseveration is relatively high and learning is low (Fig. 1h) [7, 10, 24, 25, 30]. This indicates that increased perseveration significantly contributes to the impaired reversal behaviour of mGluR5 KO mice. These data, therefore, indicate normal mGluR5 function is crucial for behavioural flexibility under conditions of low stress.

### mGluR5 KO mice show impaired performance in the touchscreen EXT task

The reversal phase of the touchscreen VDR task requires animals to exhibit behavioural flexibility by inhibiting a response driven by a previously learned stimulus-reward association while simultaneously learning a new association. To determine if the perseverative phenotype we observed in the VDR task was a more general behavioural consequence of the absence of mGluR5, we also assessed these mice in another standardized test for behavioural flexibility - the touchscreen EXT task. In this task, stimulus is no longer associated with reward and analyzing the rate of continuous responses provides a measure of the animal’s tendency to perseverate. Like the VDR, the EXT requires animals to inhibit a previously learned response behaviour, but this occurs without any need to simultaneously learn a new stimulus-reward association [16, 31].

**Fig. 2:**
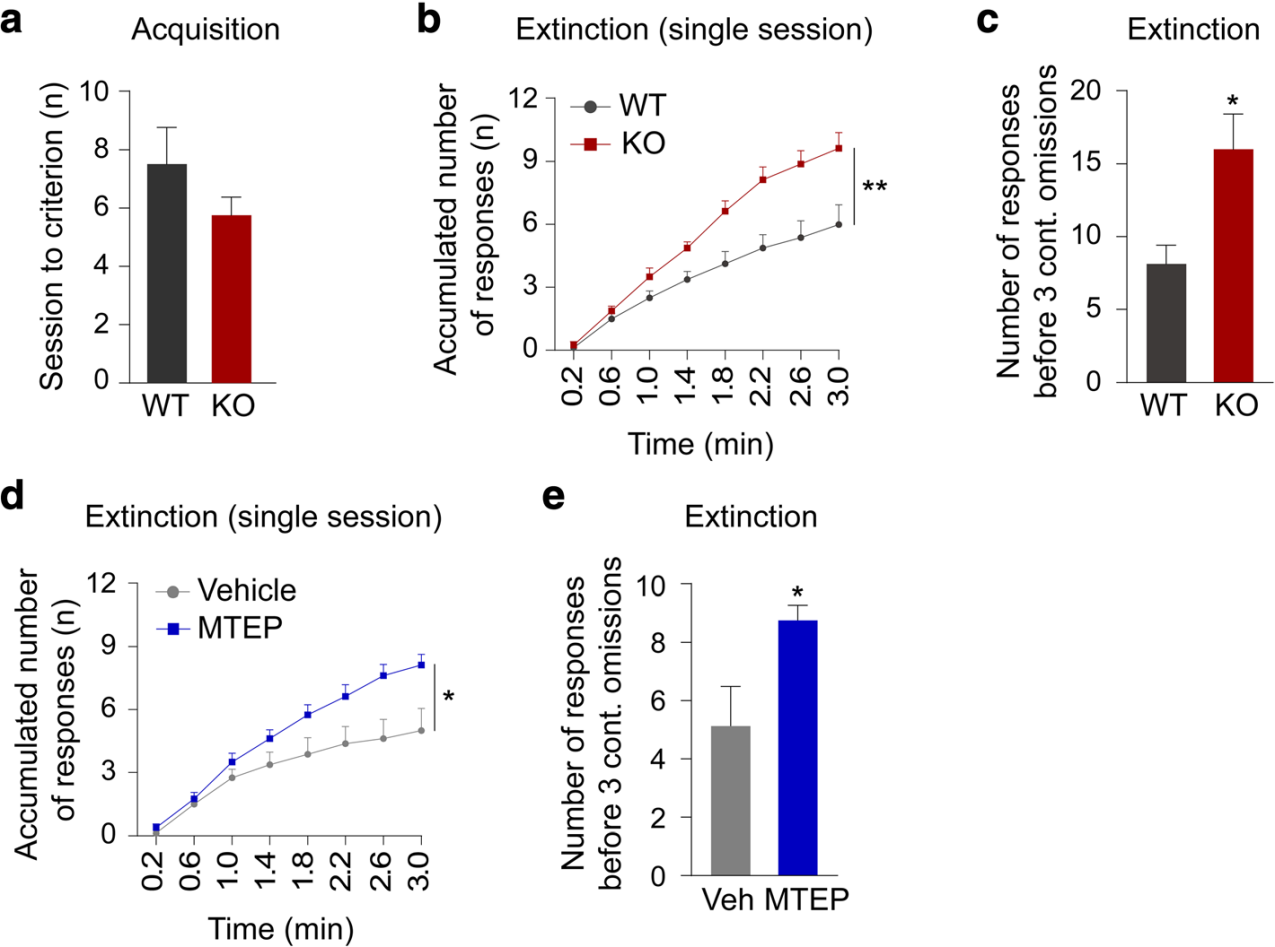
mGluR5 genetic ablation and pharmacological antagonism impair extinction performance in mice. **a** Mean number of sessions required to reach criterion (completion of 30 trials within 12.5 min for 5 consecutive days). WT group *n* = 8 and mGluR5 KO group *n* = 8. **b** Single-session analysis (1st session) of the first 3 min of extinction task (Two-way RM ANOVA, main effect of genotype; **p = 0.006). **c** Number of correct responses before making 3 consecutive omissions (Unpaired *t* test; *p = 0.012). **d** Single-session analysis (1st session) of the first 3 min of extinction task (Two-way RM ANOVA, main effect of genotype; *p = 0.032). MTEP-treated group n = 8 and vehicle-treated group n = 8. **e** Number of correct responses before making 3 consecutive omissions (Unpaired *t* test; *p = 0.027). All data are presented as means ± s.e.m.

In the acquisition phase of the EXT task, we did not detect any significant difference between WT and KO mice (Fig. 2a). In a single session analysis of extinction phase in the EXT task, however, we found mGluR5 KO mice emit a significantly higher number of stimulus responses than WT mice. This is consistent with impaired performance (Fig. 2b, two-way RM ANOVA, main effect of genotype; F(1,14) = 10.7, *p =* 0.006, main effect of time; F(7,98) = 129, *p <* 0.001, genotype x time interaction; F(7,98) = 8.82, *p <* 0.001 and Fig. 2c, unpaired *t* test; *p = 0.012) (Fig. 2b,c). Due to the rapid extinction observed with the current EXT protocol, we adopted a more stringent acquisition learning criterion to strengthen stimulus-reward association so that we could perform multi-session analyses of extinction. As we observed in the single session analyses, we observed slower extinction of stimulus-reward memories in mGluR5 KO mice than WT mice in the multi-session analyses (Additional file 1: Figure S1).

### Pharmacological antagonism of mGluR5 in C57BL/6J mice mirrors KO EXT behaviour

To determine if the phenotypes we observed in the mGluR5 KO mice are developmental effects resulting from the constitutive absence of mGluR5 expression, we treated a group of C57BL/6J mice with the mGluR5 antagonist MTEP and assessed their behaviour with the touchscreen EXT task. Like the mGluR5 KO mice, MTEP-treated animals emit significantly more stimulus responses in the extinction phase of EXT relative to vehicle-treated controls (Fig. 2d, two-way RM ANOVA, main effect of treatment; F(1,14) = 5.64, *p =* 0.032, main effect of time; F(7,98) = 73.8, *p <* 0.001, treatment x time interaction; F(7,98) = 4.97, *p <* 0.001 and Fig. 2e, unpaired *t* test; *p = 0.027) (Fig. 2 d, e). This suggests the behavioural effects we observed in the mGluR5 KO animals are not due to any neurodevelopmental alterations resulting from their genetic manipulation.

### mGluR5 KO performance on touchscreen FR and PR schedules is consistent with elevated perseveration

PR schedules that require increasing numbers of responses for a reinforcer over successive sessions are commonly used to assess behavioural motivation [15, 23, 32]. They are sensitive, however, to “non-motivational” influences such as perseverative behaviour [33, 34, 35]. Given the perseverative responses observed in the mGluR5 KO mice in both the VDR and EXT tasks, we used the touchscreen FR and PR tasks to determine if these animals exhibit perseverative behaviour in a context in which response inhibition is not explicitly required for successful performance.

**Fig. 3:**
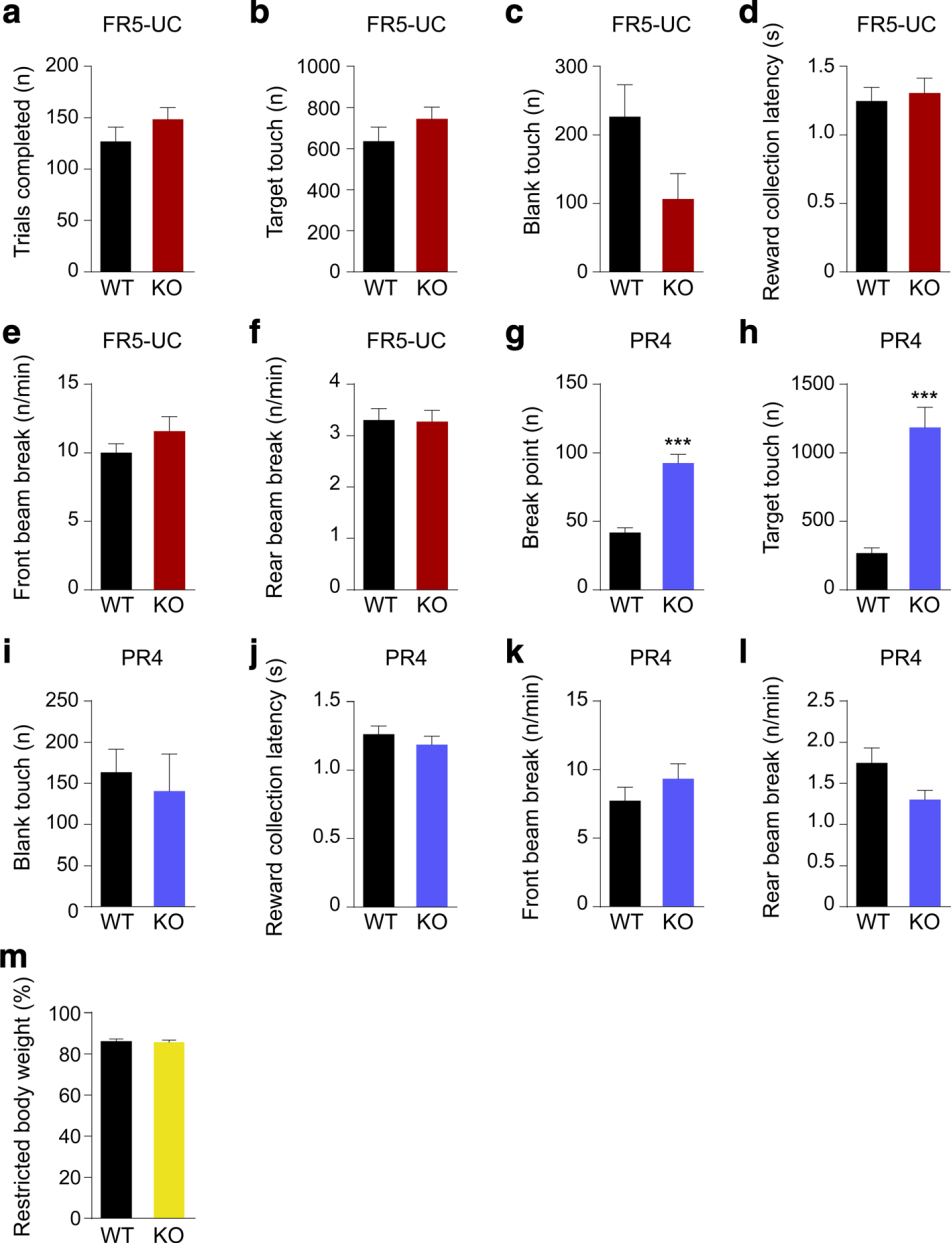
mGluR5 KO mice exhibit elevated breakpoints relative to WT littermates in touchscreen PR4 schedule. **a** Number of trials completed in FR5-UC. **b** Target touches in FR5-UC (Total number of responses at the target screen location). **c** Blank touches in FR5-UC (Total number of responses at the 4 non-target screen locations). **d** Reward collection latency during FR5-UC (Time between completion of the final target touch of a trial and entry to the reward magazine for reward collection). **e-f** Rate of IR beam breaks in the front and rear zones of the touchscreen apparatus during FR5-UC. **g** Breakpoint in PR4 (Number of target responses emitted by an animal in the last successfully completed trial, before session termination or 60 min time-out). **h** Target touches in PR4. **i** Blank touches in PR4. **j** Reward collection latency during PR4. **k**-**l** Rate of IR beam breaks in the front and rear zones of the touchscreen apparatus during PR4. **m** Percentage of maintained restricted body weight relative to the baseline free-feeding weight. WT group *n* = 10 and mGluR5 KO group *n* = 10, unpaired *t* test; ***p < 0.001. All data are presented as means ± s.e.m.

Following training through FR1, 2, 3, and performance stabilization in the more challenging FR5 schedule with 30 trials per 60-min session, we evaluated the mice in an ‘uncapped’ FR5 session (FR5-UC) lacking the 30-trial performance limit. We chose this FR5-UC schedule to permit operant responding to be expressed under conditions of a moderate and consistent work requirement. This should have feasibly allowed mice to work to the point of satiation. Under the FR5-UC schedule, we did not observe any differences in performance between genotypes (Fig. 3 a-f), suggesting that mGluR5 KO mice do not show different levels of satiety compared to wild-type mice.

Following the FR5-UC schedule, we evaluated the mice on a PR4 schedule in which the number of responses required to earn a single reward was linearly increased by four in each subsequent trial. This results in mice being required to both emit more responses to earn the same reinforcer but also to continue to emit responses for increasingly longer periods before receiving reinforcement. We found the mGluR5 KO group achieve a significantly higher breakpoint and total number of correct touches than the WT control group in the PR4 schedule (Fig. 3g, h) with no significant difference in blank touches, reward collection latency, or front and rear beam break rate (Fig. 3i, j, k, l). We compared body weights between genotypes as a potential confounding effect on FR and PR performance, but we found no significant between-group differences in % restricted body weight (Fig. 3m).

We also evaluated mice subjected to the touchscreen EXT task on a PR4 schedule to examine whether a single cohort of animals could be assessed on multiple tasks sequentially. We found a similar pattern of results to animals only trained previously on ratio schedules (Fig. 3g) in that mGluR5 KO mice exhibited elevated breakpoints in the PR4 schedule relative to WT animals after completing the EXT task (Additional file 2: Figure S2). This finding supports the validity of assessing rodents in the touchscreen apparatus using ‘batteries’ of multiple tasks that target distinct cognitive domains. In this case, prior completion of the touchscreen EXT task had no impact on performance of touchscreen ratio schedules. This demonstrates the viability of sequential assessment of cognitive flexibility, motivation and perseverative behavior.

**Fig. 4:**
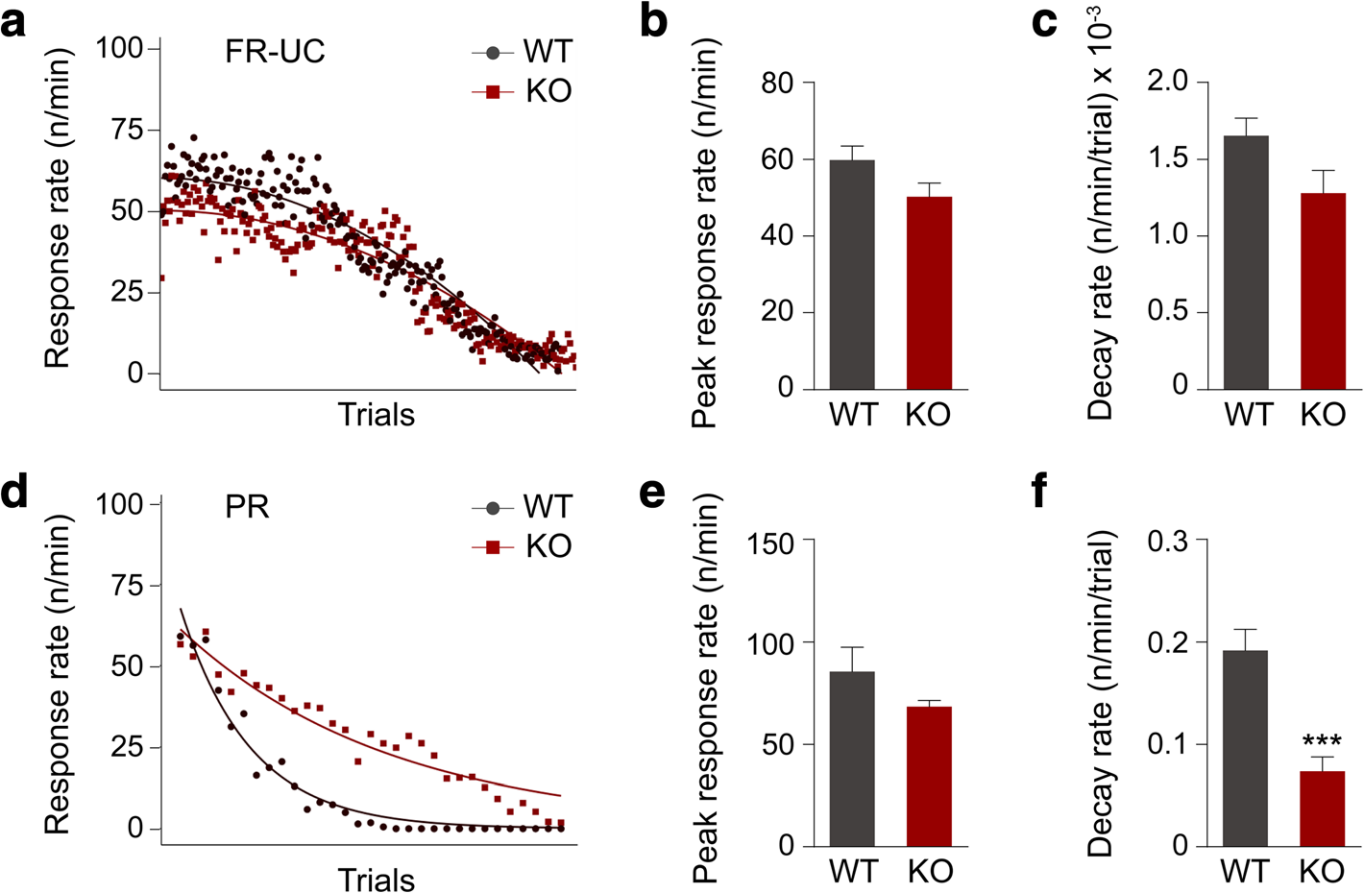
Within-session response rate analysis in FR5-UC and PR4. **a** Changes in response rate in the first session of FR5-UC. **b** Peak response rate in FR5-UC. **c** Decay rate in FR5-UC. **d** Changes in response rate in the first session of PR4. **e** Peak response rate in PR4. **f** Decay rate in PR4*.* WT group *n* = 10 and mGluR5 KO group *n* = 10, unpaired *t* test; ***p < 0.001. All data are presented as means ± s.e.m.

We also found via a within-session response rate analysis [21, 26, 27] for the first session of FR5-UC (Fig. 4a, b, c) and PR4 (Fig. 4d, e, f) that in both schedules, while there is no effect of genotype on predicted maximum or peak response rate (Fig. 4 b, e), the response decay rate for mGluR5 KO mice is significantly lower than that of the WT control group in PR4 (Fig. 4f), but not in FR5-UC (Fig. 4c). This emphasizes that the initial motivation to obtain reward is consistent between groups but that, in spite of time-dependent changes in the conditions that would arguably weaken the stimulus-reward association in the PR schedule (e.g. increasing time since last reward, increasing effort expenditure required per reward), mGluR5 KO mice persist in responding considerably more than WT littermates. Although mice typically show a much faster response rate decay in PR4 compared to FR, the mGluR5 KO mice appear to be less sensitive to the instrumental extinction process because they show more perseverative behaviour. This perseverative behaviour is independent of satiety and initial motivation for reward.

### mGluR5 KO mice exhibit altered effort-related decision making in the ERC task

**Fig. 5:**
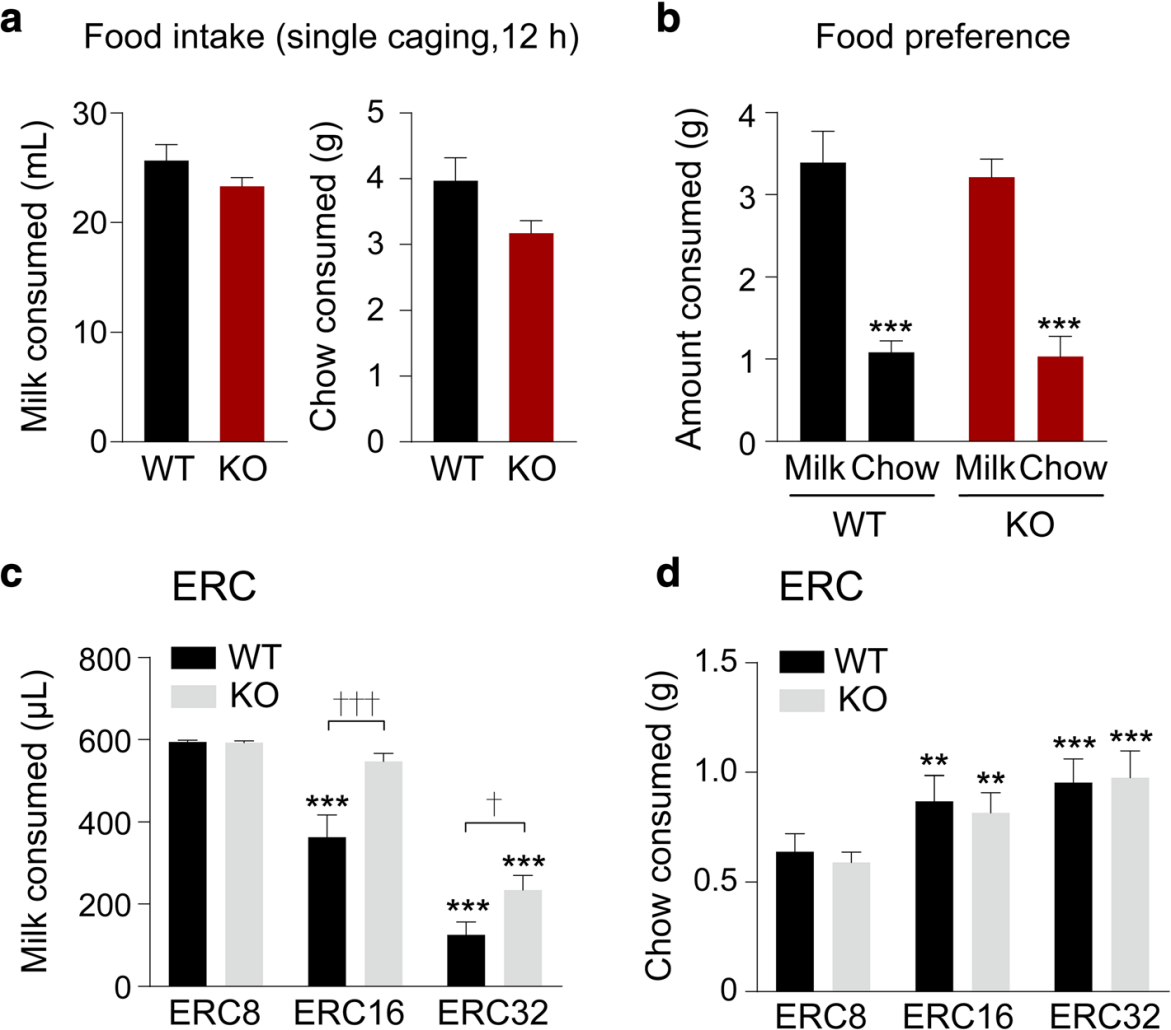
Profiles of food intake and effort-related choice behaviour of mGluR5 KO mice and WT littermates. **a** Consumption of freely available standard laboratory chow and strawberry milk (given in isolation) were measured for 12 h. WT group *n* = 10 and mGluR5 KO group *n* = 10. **b** Comparison of freely available SM and chow consumption measured for an hour a day for 5 consecutive days under maintained food restriction conditions. WT group *n* = 6 and mGluR5 KO group *n* = 6 (Two-way ANOVA, main effect of chow or SM consumption; ***p < 0.001). (**c**-**d)** WT group *n* = 10 and mGluR5 KO group *n* = 10 were analyzed for ERC performance. **c** SM consumption across increasing operant work requirements in the ERC task (Two-way RM ANOVA followed by Bonferroni post hoc test, †*p =* 0.044, †††*p <* 0.001 between genotypes, ***p < 0.001 compared to the ERC8 condition). **d** Chow consumption across increasing operant work requirements in the ERC task (Two-way RM ANOVA followed by Bonferroni post hoc test, **p = 0.002, ***p < 0.001 compared to the ERC8 condition). All data are presented as means ± s.e.m

We first evaluated reward preferences in mGluR5 KO and WT littermates in a single-cage food (chow or SM) consumption test and a chow vs. SM preference test to ensure that both groups had similar satiety levels and assign similar relative values to two reinforcers [15]. We found no difference between genotypes in the amounts of freely available SM and chow consumed (Fig. 5a) or in the preference for SM over chow (Two-way ANOVA, main effect of genotype; F(1,20) = 0.191, *p* = 0.667, main effect of condition; F(1,20) = 72.6, *p* < 0.001, genotype x condition interaction; F(1,20) = 0.0548, *p* = 0.817) (Fig. 5b). These results suggest comparable satiation thresholds and hedonic valuation. This is also consistent with our previous finding of no change in the hedonic responses of mGluR5 KO mice towards sucrose in a non-stress condition [13].

In the ERC assessment, which is used to measure animal reward-decision making, standard laboratory chow pellets were scattered randomly across the floor of each chamber. We compared the amount of SM consumed through touchscreen responding to the amount of freely available chow consumed using a range of touchscreen work requirements (ERC8, 16, 32; eight, sixteen and thirty-two touchscreen responses per SM delivery). As expected, increasing the work requirement resulted in a progressive decrease in touchscreen responding for SM in both genotypes (Two-way RM ANOVA, main effect of genotype; F(1,18) = 8.49, *p =* 0.009, main effect of operant work requirement; F(2,36) = 153, *p <* 0.001, genotype x operant work requirement interaction; F(2,36) = 7.61, *p =* 0.002) (Fig. 5c). Correspondingly, both genotypes progressively increased the consumption of standard chow as the work requirement increased (Two-way RM ANOVA, main effect of genotype; F(1,18) = 0.042, *p =* 0.839, main effect of operant work requirement; F(2,36) = 34.9, *p <* 0.001, genotype x operant work requirement interaction; F(2,36) = 0.486, *p =* 0.619) (Fig. 5d).

Critically, at both ERC16 and ERC32, we found mGluR5 KO mice consume significantly more SM (and so emit significantly more touchscreen responses) than WT (Fig. 5c). It would therefore be reasonable to expect that mGluR5 KO mice would consume less freely available chow relative to WT animals. However, under ERC 16 and ERC 32 conditions, we found mGluR5 KO mice consume similar amounts of chow compared to WT mice (Fig. 5d). Together, these data suggest that mGluR5 KO mice tend toward making perseverative operant responses, even in the presence of an opportunity to make an effort-based decision.

## Discussion

mGluR5 is ubiquitously expressed across several key brain areas, such as the hippocampus, nucleus accumbens, dorsal striatum, and cerebral cortex [36, 37]. It also plays a critical role in various forms of synaptic plasticity [1, 22, 38–40]. These characteristics have suggested mGluR5 as an important therapeutic target for the treatment of various neuropsychiatric and neurodegenerative disorders [41–43], with the potential for alleviating problems associated with disruptions across a number of important cognitive domains.

One such cognitive domain is behavioural flexibility, which is critical to the success of an organism exposed to a changing environment and its associated alterations in stimulus-reward associations. Response inhibition is a key process required for effective behavioural flexibility, and a variety of studies have reported impairments in response inhibition in mice deficient in mGluR5 either because of a genetic deletion or because of pharmacological antagonism [1–5].

However, most of the studies that have implicated mGluR5 in behavioural flexibility have assessed genetically or pharmacologically manipulated animals under high stress conditions, for example using classical (Pavlovian) fear conditioning or shock avoidance paradigms and forced swim paradigms [1–5]. As stress-exposure typically affects cognition adversely (although sometimes facilitates cognitive functions) [6–10], it is challenging to separate the effect of a manipulation on behavioural flexibility from its effects on global affective state under high stress conditions. Indeed, this is of particular significance because of mGluR5’s critical role in resilience and in the responses of animals to stressful stimuli [13, 14]. To overcome this limitation, here we leveraged the benefits of the rodent touchscreen cognitive assessment apparatus [16, 18, 20] to evaluate behavioural flexibility in the mGluR5 KO mouse model under low stress conditions using a battery of appetitive reinforced behavioural tasks.

In the two-choice VDR task, we did not detect any differences in perceptual discrimination ability, or in the acquisition of the required stimulus-response associations. This is in contrast to previous studies that have suggested mGluR5 KO mice exhibit impaired acquisition in both fear conditioning and the Morris water maze [1, 44]. This discrepancy may stem from differences in the behavioural tasks themselves, such as the greater spatial demands inherent in the Morris water maze, but it also may be affected by the considerably higher levels of stress associated with these procedures.

In contrast, we did find a substantial impairment in mGluR5 KO mice when performing the reversal phase of the VDR task. This is consistent with their impaired reversal of spatial learning, as observed in the Morris water maze [1]. Interestingly, further analysis of the VDR data revealed that this reversal impairment was driven at least in part by a significant increase in the perseverative (early) epoch [7, 10, 24, 25] of the process in the KO animals.

To determine if this perseverative phenotype is generalized beyond tasks in which both response inhibition and stimulus-reward learning are required [16, 31], we assessed mGluR5 KO and WT littermates in the touchscreen EXT task. This also revealed an impairment in the mGluR5 KO animals, with this group emitting significantly more responses to the touchscreen stimulus, despite a lack of reward for this behaviour. This is also consistent with the extinction deficit previously observed in mGluR5 KO mice in both contextual and cued fear conditioning [1]. We now extend this finding to non-aversive paradigms. Replication of this phenotype in WT mice treated with the mGluR5 antagonist MTEP and assessed in EXT indicates this behavioural effect of mGluR5 KO cannot be attributed to any neurodevelopmental alteration induced by the genetic manipulation.

The ratio schedule we used here was designed to study motivation [23, 32, 45]. The canonical interpretation of elevated breakpoints is increased motivation. Thus, the higher breakpoints achieved by mGluR5 KO mice indicate that their deficits in reversal learning and EXT are unlikely due to a lack of motivation. Moreover, several studies have shown that PR breakpoints are sensitive to perseveration [33–35]. The careful analysis we performed in this study revealed that the high PR breakpoints we observed for the mGluR5 KO mice were due to perseveration.

Interestingly, the elevated performance we observed in these ratio schedule tasks conflicts with studies of food-maintained PR performance in rats acutely administered with the mGluR5 antagonists MPEP and MTEP. These compounds cause either a suppression in performance or no effect [46–48]. These differences may be attributable to species differences, differences in the details of the behavioural assessment paradigm, or differences in the mGluR5 manipulation approach producing off-target/side-effects.

In the final element of the touchscreen-based behavioural characterization presented here, we evaluated the performance of the mGluR5 KO animals in the touchscreen ERC paradigm. This assessment is similar to the FR and PR schedules, but it provides animals with the choice of emitting responses to obtain a preferred reward or consuming a less preferred but freely available alternative. This analysis revealed that while mGluR5 KO mice exhibit a similar pattern of choice behaviour to WT by numerically increasing consumption of the less preferred reward as the work requirement associated with the more preferred reward rises, at the higher work requirements (ERC16 and ERC32), the mGluR5 KO mice also emit significantly more touchscreen responses than WT littermates. Interestingly, we did not observe any difference in touchscreen responses at the relatively less challenging ERC8 work requirement. This is reminiscent of the similar levels of initial response rate to obtain reward between WT and mGluR5 KO animals that we revealed in the within-session response rate analysis for PR4 (Fig. 4d, e, f). These data again support the hypothesis that mGluR5 KO promotes perseverative responses rather than increasing motivation, particularly under conditions of high effort expenditure (operationally defined by the number of responses required per reward) or extended delay between reinforcer deliveries.

It is notable that the degree of perseveration exhibited by the mGluR5 KO may be insufficient to completely disrupt the cost-benefit decision making required in the ERC task. In the canonical ERC procedure, when lever presses are reduced, the animals show a compensatory relocation of behaviour toward the low effort/low reward option. While milkshake consumption by the mGluR5 KO mice is higher relative to WT at ERC16 and ERC32, chow consumption relative to WT did not change at any work requirement. Coupled with the matched SM vs. chow preference of the genotypes, this suggests that the KO animals consumed more milkshake because they show increased perseveration rather than because they actively allocated more response resources to consume milkshake over standard laboratory chow.

While this paper was in preparation, another group independently reported similar phenotypes of the mGluR5 KO mice in touchscreen-based VDR and EXT cognitive tests [49]. The fact that two independent groups are reporting similar data support the reliability of the observed cognitive phenotypes of mGluR5 KO mice and the high reproducibility of automated touchscreen-based cognitive test methods. The only discrepancy between the two studies is that we did not find evidence that mGluR5 KO impairs the acquisition of stimulus-reward association in the visual discrimination task in our study. Both groups used mice with the same genetic background. While Zeleznikow-Johnston et al. [49] used correction trials in their visual discrimination test, we did not in this study. It is possible that the correction trials are more efficient feedback to wild-type mice compared with mGluR5 KO mice to counteract the development of side or stimulus biases. This would enhance acquisition learning in wild-type mice. This can be tested in the future with more precise experiments comparing acquisition learning in the absence or presence of correction trials.

Here we have assessed the role of mGluR5 in behavioural flexibility in a low stress, appetitively reinforced context using tasks delivered via the rodent touchscreen assessment system. We found that mGluR5 KO mice have deficits in behavioural flexibility that are manifested as an impaired capacity to reverse in a two-choice VDR assay and an impaired capacity to extinguish a previously acquired operant response. The impaired behavioral flexibility of mGluR5 KO mice is driven by perseverative behavior. Because perseveration occurs in several disease states (i.e., epilepsy, dementia, schizophrenia, and stroke), patients suffering from these diseases may benefit from pharmacological modulation of mGluR5 activity. This is all the more promising because a positive allosteric modulator of mGluR5 was found to reduce perseverative behavior in a schizophrenia mouse model [50]. Several brain regions including the orbitofrontal cortex, infralimbic cortex and amygdala are thought to be responsible for behavioural flexibility in humans and rodents [51–54]. Here, the deficits in mGluR5 KO flexibility can be attributed to high levels of perseverative responding, which have often been associated with orbitofrontal dysfunction [53, 55–58]. Further studies will help clarify how mGluR5 regulates perseverative responding, which regions of the brain are involved, and how behavioural flexibility can be normalized in mGluR5-deficient brains.

## Additional files

Additional file 1: **Figure S1.** mGluR5 KO mice emitted more stimulus responses than WT littermates in the EXT task over multiple sessions. Multi-session analysis of the percentage of responses during extinction task. WT group *n* = 8 and mGluR5 KO group *n* = 9, Two-way RM ANOVA, main effect of genotype F(1,15) = 10.5; *p* = 0.005, main effect of session; F(9,135) = 23.3, *p <* 0.001, genotype x session interaction; F(9,135) = 3.1, *p =* 0.002, followed by Bonferroni post hoc test, **p = 0.001 between genotypes for session 1,2 and **p = 0.002 between genotypes for session 3. All data are presented as means ± s.e.m. (DOCX 846 kb)

Additional file 2: **Figure S2.** mGluR5 KO mice perform better than WT littermates in the PR4 schedule task evaluated after undergoing the touchscreen EXT task. **a** Breakpoint in PR4. **b** Target touches in PR4. **c** Blank touches in PR4. WT group *n* = 10 and mGluR5 KO group *n* = 10, unpaired *t* test; ***p < 0.001. All data are presented as means ± s.e.m. (DOCX 2097 kb)

## Abbreviations

ANOVA: Analysis of variance
ERC: Effort-related choice
EXT: Extinction
FR: Fixed ratio
FR-UC: Uncapped fixed ratio
IR: Infra-red
ITI: Inter-trial interval
KO: Knockout
LED: Light-emitting diode
mGluR5: metabotropic glutamate receptor 5
MPEP: 2-Methyl-6-(phenylethynyl)pyridine
MTEP: 3-((2-methyl 1,3-thiazol-4-yl)ethynyl) pyridine
PR: Progressive ratio
RM: Repeated measures
SEM: Standard error of the mean
SM: Strawberry-flavored milk
VDR: Visual discrimination-reversal
WT: Wild-type
