# Supplementary material for: Assessment of mGluR5 KO mice under conditions of low stress using a rodent touchscreen apparatus reveals impaired behavioural flexibility driven by perseverative responses
Source: Mol Brain. 2019 Apr 11;12:37. doi: 10.1186/s13041-019-0441-8 (PMC6458840; doi:10.1186/s13041-019-0441-8)
Supplement: Supplementary file 1 — Figure S1. mGluR5 KO mice emitted more stimulus responses than WT littermates in the EXT task over multiple sessions. Multi-session analysis of the percentage of responses during extinction task. WT group n = 8 and mGluR5 KO group n = 9, Two-way RM ANOVA, main effect of genotype F(1,15) = 10.5; p = 0.005, main effect of session; F(9,135) = 23.3, p < 0.001, genotype x session interaction; F(9,135) = 3.1, p = 0.002, followed by Bonferroni post hoc test, **p = 0.001 between genotypes for session 1,2 and **p = 0.002 between genotypes for session 3. All data are presented as means ± s.e.m. (DOCX 846 kb) [file 13041_2019_441_MOESM1_ESM.docx]

**
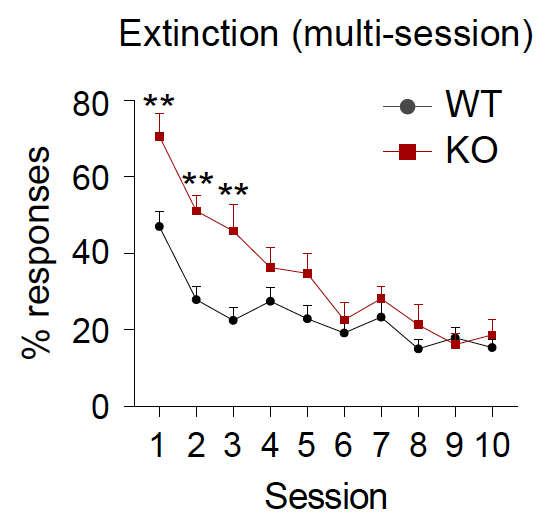
**

**Figure S1**. mGluR5 KO mice emitted more stimulus responses than WT littermates in the EXT task over multiple sessions. Multi-session analysis of the percentage of responses during extinction task. WT group n = 8 and mGluR5 KO group n = 9, Two-way RM ANOVA, main effect of genotype F(1,15)=10.5; *p* = 0.005, main effect of session; F(9,135) = 23.3, *p <* 0.001, genotype x session interaction; F(9,135) = 3.1, *p =* 0.002, followed by Bonferroni *post* *hoc* test, ***p =* 0.001 between genotypes for session 1,2 and ***p =* 0.002 between genotypes for session 3. All data are presented as means ± s.e.m.
