# Supplementary material for: Assessment of mGluR5 KO mice under conditions of low stress using a rodent touchscreen apparatus reveals impaired behavioural flexibility driven by perseverative responses
Source: Mol Brain. 2019 Apr 11;12:37. doi: 10.1186/s13041-019-0441-8 (PMC6458840; doi:10.1186/s13041-019-0441-8)
Supplement: Supplementary file 2 — Figure S2. mGluR5 KO mice perform better than WT littermates in the PR4 schedule task evaluated after undergoing the touchscreen EXT task. a Breakpoint in PR4. b Target touches in PR4. c Blank touches in PR4. WT group n = 10 and mGluR5 KO group n = 10, unpaired t test; ***p < 0.001. All data are presented as means ± s.e.m. (DOCX 2097 kb) [file 13041_2019_441_MOESM2_ESM.docx]

**
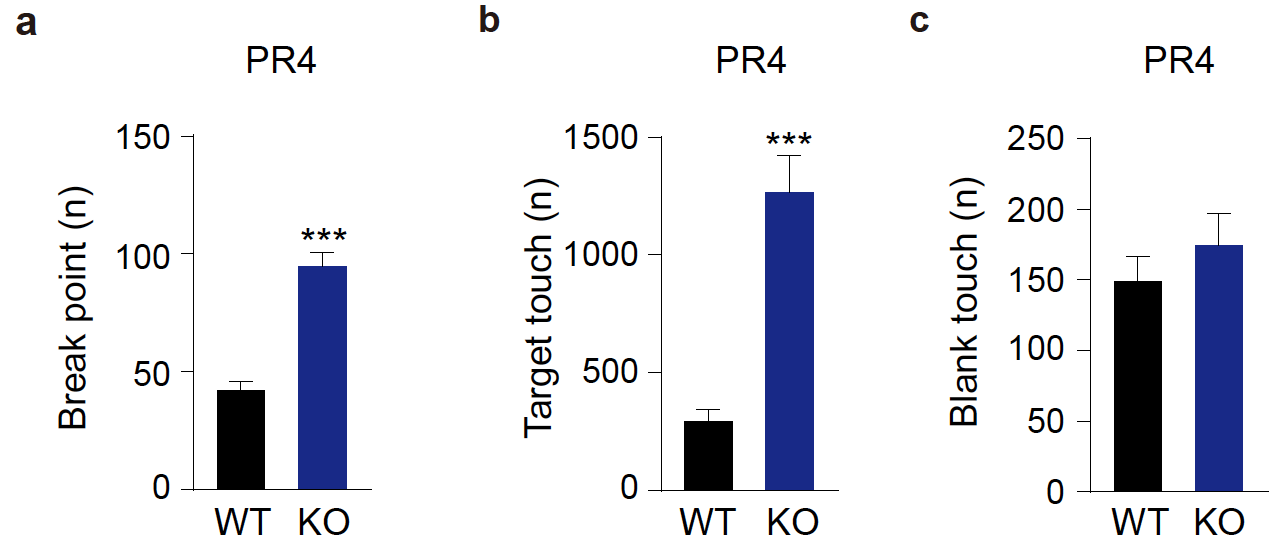
**

**Figure S2**. mGluR5 KO mice perform better than WT littermates in the PR4 schedule task evaluated after undergoing the touchscreen EXT task. **a** Breakpoint in PR4. **b** Target touches in PR4. **c** Blank touches in PR4. WT group n=10 and mGluR5 KO group n=10, unpaired *t* test; ****p* < 0.001. All data are presented as means ± s.e.m.
